# Functional and Radiographic Outcomes of Custom Acetabular Implants for Severe Acetabular Defects: A Trauma Unit Perspective

**DOI:** 10.7759/cureus.86987

**Published:** 2025-06-29

**Authors:** Geeth Silva, Djon Lopez, Philip Sloper, Nicholas Wardle

**Affiliations:** 1 Trauma and Orthopaedics, Colchester General Hospital, Colchester, GBR

**Keywords:** custom-made implants, major trauma, orthopaedic traumatology, orthopedic implant related infection, total hip athroplasty

## Abstract

Introduction

Increasing total hip replacements means reconstructive surgeons face more complex challenges, including acetabular bone loss, repeat revisions and complex pathologies. Paprosky type 3 defects and pelvic discontinuities (PDs) need careful planning, specialised tools and a high degree of skill. Literature has yet to agree on the most optimal way to manage these challenges, with the birth of custom acetabular implants (CAIs) to treat severe acetabular defects representing a reliable solution. However, their integration into common practice is still evolving. This study evaluated clinical and radiographic outcomes of CAIs, analysing patient demographics and surgical indications associated with their use.

Methods

This single-centre retrospective review at Colchester General Hospital assessed acetabular reconstruction using a custom-made 3D-printed titanium implant between 2018 and 2024.

Outcome measures included implant survivorship, complications (dislocation, fracture, infection), radiographic satisfaction (assessing for loosening or migration of the implant), and clinical outcome identified through mobility postoperatively and the Oxford Hip Score.

Results

Sixteen patients were enrolled in the study. Sixty-three percent (63%) achieved full weight bearing, and 94% achieved satisfactory radiographic outcomes. There was a 19% complication rate, with two infections and one dislocation. Patients chosen for the procedure had a Charlson Comorbidity Index (CCI) of 0.5 and an average age of 73, resulting in no postoperative admissions to the intensive care unit, thus displaying their capacity to withstand the surgery.

Discussion

CAIs are useful in challenging cases with PD and acetabular bone loss requiring only a posterior approach. 3D reconstructions aid both planning and strategising, thus avoiding future complications. CAIs are useful in these salvage cases with limited options; however, candidates chosen need to be fit enough to tolerate the surgery. Providing such a service needs support from the parent company alongside careful follow-up for at least 12 months. Overall, CAIs are advocated to treat complex acetabular defects and PDs to improve patient function and quality of life.

## Introduction

Primary total hip arthroplasty (THA) is regarded as one of the most successful interventions due to improved quality of life and reduced pain, which patients benefit from [1]. Although implants can last up to 20 years, complications, including infection, aseptic loosening and instability, can lead to their failure [2]. Moreover, with the increasing presence of THAs in the patient population, reconstructive surgeons face more complex challenges, including acetabular bone loss, repeat revisions and complex pathologies. These complications can lead to severe bone loss, as classified by the Paprosky system.

The classification ranges from type 1 (minimal bone loss) to type 3, with type 3B (>60% bone loss with substantial superomedial migration of the hip centre) being the most severe [3]. Such defects and pelvic discontinuities (PDs) need careful planning within the multiple disciplinary team (MDT), specialised tools and a high degree of skill. Previously, 3B type defects and pelvic discontinuities were managed using a jumbo cup and acetabular cages.

However, the current literature has yet to agree on the most optimal way to manage these challenges. A systematic review showed that cup cages and highly porous shells had over 90% survival rates. Still, there was no consensus on how different types of acetabular reconstruction methods optimised the healing potential of PDs [4]. The birth of custom acetabular implants (CAIs) to treat severe acetabular defects represents a reliable solution [5]. However, their integration into common practice is still evolving, with only one study in the U.K. recording their outcomes [6].

This study aimed to assess the clinical and radiographic results associated with using custom-made 3D-printed acetabular cups for complex acetabular defects over a six-year time horizon. Moreover, we discuss the patient demographics and the context in which these implants are most suitable for use.

## Materials and methods

This single-centre study was conducted at Colchester General Hospital over six years. The team retrospectively reviewed our database to identify all patients who had undergone acetabular reconstruction with a custom-made 3D-printed titanium implant with or without pelvic discontinuity between 2018 and 2024 (Appendix).

Each patient gave consent according to local ethical guidelines. The hip arthroplasty team at Colchester General Hospital performed surgical treatment and follow-up evaluations. 

Inclusion and exclusion criteria

Patients were included in the study if they had a failed acetabular implant following a primary THA, had undergone multiple previous revision procedures, and were discussed at an MDT meeting where a custom implant was deemed the most appropriate option. All included cases involved a Paprosky type 3B acetabular defect (severe form of bone loss characterised by superomedial implant migration, poor bone stock) with or without pelvic discontinuity [3].

Patients were excluded if they required concurrent surgical interventions unrelated to the acetabular reconstruction or if they were considered medically unfit to undergo further revision surgery.

Outcome measures

The primary outcome measure was implant survivorship. Secondary outcomes included the incidence of complications (dislocation, periprosthetic fracture, or infection) and radiographic satisfaction as through assessing for implant loosening or migration. 

Surgical workflow 

Potential patients were either referred to the team through a clinical review or the acute on-call team. After a clinical and radiological assessment of their condition, their case would be discussed in the Hip MDT. This MDT involves Reconstructive Hip Consultants alongside microbiologists and radiologists. Therefore, the most optimal treatment method would be decided and documented before relaying the information to the patient. 

**Figure 1:**
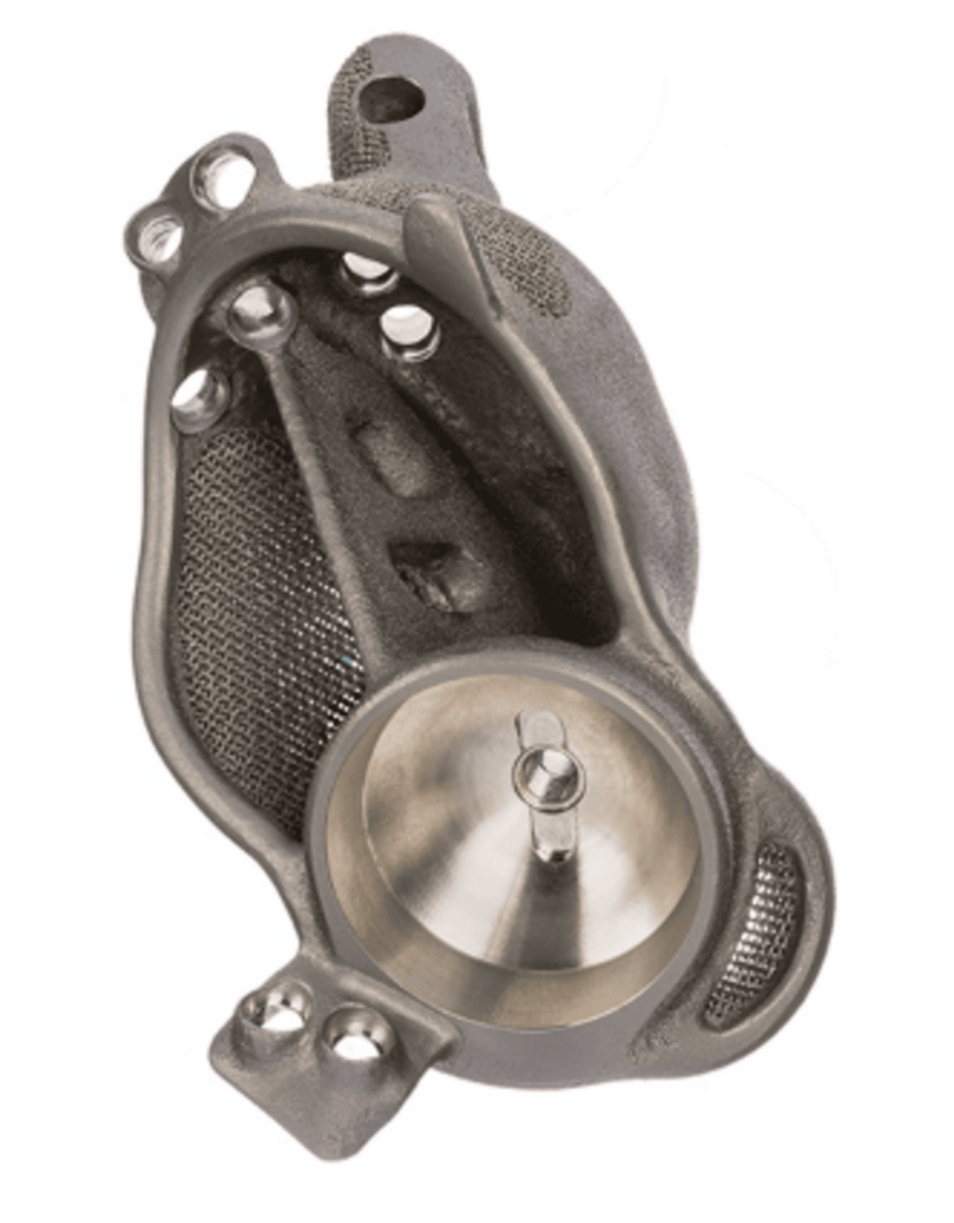
Custom Acetabular Implant: ProMade; LimaCorporate Image used with the permission of LimaCorporate [7]

After obtaining the patient’s CT Hip, the images are sent to the contracted company (e.g. ProMade; LimaCorporate, San Daniele del Friuli, Italy), which can then utilise the capabilities of 3D printing to create patient-specific solutions for complex acetabular reconstructions (Figure 1). The implant is titanium to closely match the Young’s modulus of bone with a trabecular titanium expansion to allow more natural load transfer and associated bone health [7].

Clinical outcomes

The supervising consultant reviewed the patient postoperatively and during the inpatient stay for early postoperative complications before discharge. The GP would review the patient for a wound check for two weeks before the supervising clinician reviews the patient six weeks later to assess clinical function. Subsequent reviews of the patient would be directed by the supervising clinician. However, usually, practice is a review at six months and then discharge at 12 months with a patient-initiated follow-up if no concerns were raised. 

The database was reviewed for mobility documentation and any complications to gauge functional improvements. Patients at baseline had limited mobility, generally restricted to a frame or wheelchair, and were deemed salvage cases. Furthermore, the patients were assessed for age, comorbidities and contralateral hip arthroplasty. Specifically, the Charlson Comorbidity Index (CCI) was used to quantify patients’ comorbidity burden. The CCI assigns weighted scores to various comorbid conditions based on the risk of mortality thus generating a cumulative score for each patient. A higher CCI indicates a greater burden of comorbid disease and predicts a higher risk of mortality [8].

Finally, patients were contacted postoperatively via telephone consultation during the time of the study to conduct a retrospective Oxford Hip Score (OHS) of their function preoperatively and postoperatively. This validated tool consists of 12 questions relating to pain and physical function, each scored from 0 to 4, yielding a total score ranging from 0 to 48. Higher scores indicate better hip function and less pain, with scores interpreted as follows: 0-19 suggests severe hip problems, 20-29 moderate, 30-39 mild, and 40-48 satisfactory joint function [9].

Radiographic outcomes 

Radiographs and CT images were assessed pre-operatively and compared to postoperative radiographs. Radiographs were taken after the operation, at six months, 12 months, and then annually thereafter. Therefore, radiographs were reviewed for signs of radiolucency, implant stability, migration, and congruency. Furthermore, positive factors such as bony ingrowth and component integrity were evaluated and correlated with clinical documentation. Specific radiographic complications can be seen in Table 1 [10-13].

**Table 1:** Radiographic assessment of Custom Prosthesis

Radiographic Complication	Radiographic Criteria
Pelvic Discontinuities	Evaluated using the ilioischial lines and the iliopectineal lines on radiographs or radiological report when CT scan performed.
Acetabular Migration	Considered to be significant when >10mm due to increased risk of complications.
Implant Loosening	Complete radiolucent line more than one millimetre in width at the bone-cement interface or any migration or tilting of the component.

Statistical analysis

Descriptive statistics were used to summarise patient demographics and baseline characteristics. As the data was non-parametric, a Wilcoxon signed-rank test was used to compare preoperative and postoperative OHS scores. A p-value of less than 0.05 was considered statistically significant. Statistical analysis was performed using the Python Pandas package. Graphs were produced using the Excel software for macOS (Microsoft, Redmond, WA, USA). 

Source of funding

This work did not receive grants from funding agencies in the public, commercial, or nonprofit sectors.

## Results

Patient evaluation 

**Figure 2:**
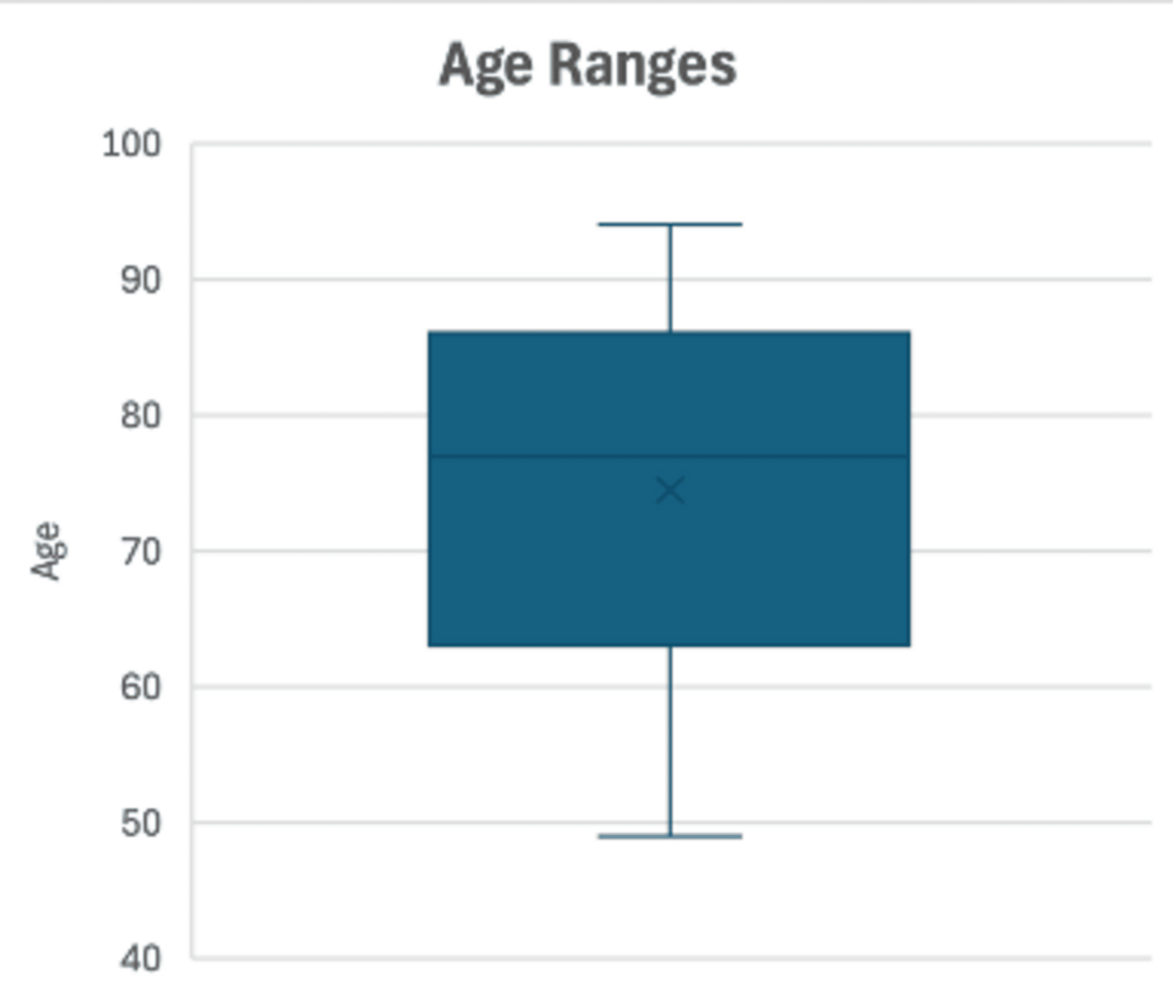
Box Chart of Age Range of Cohort

**Figure 3:**
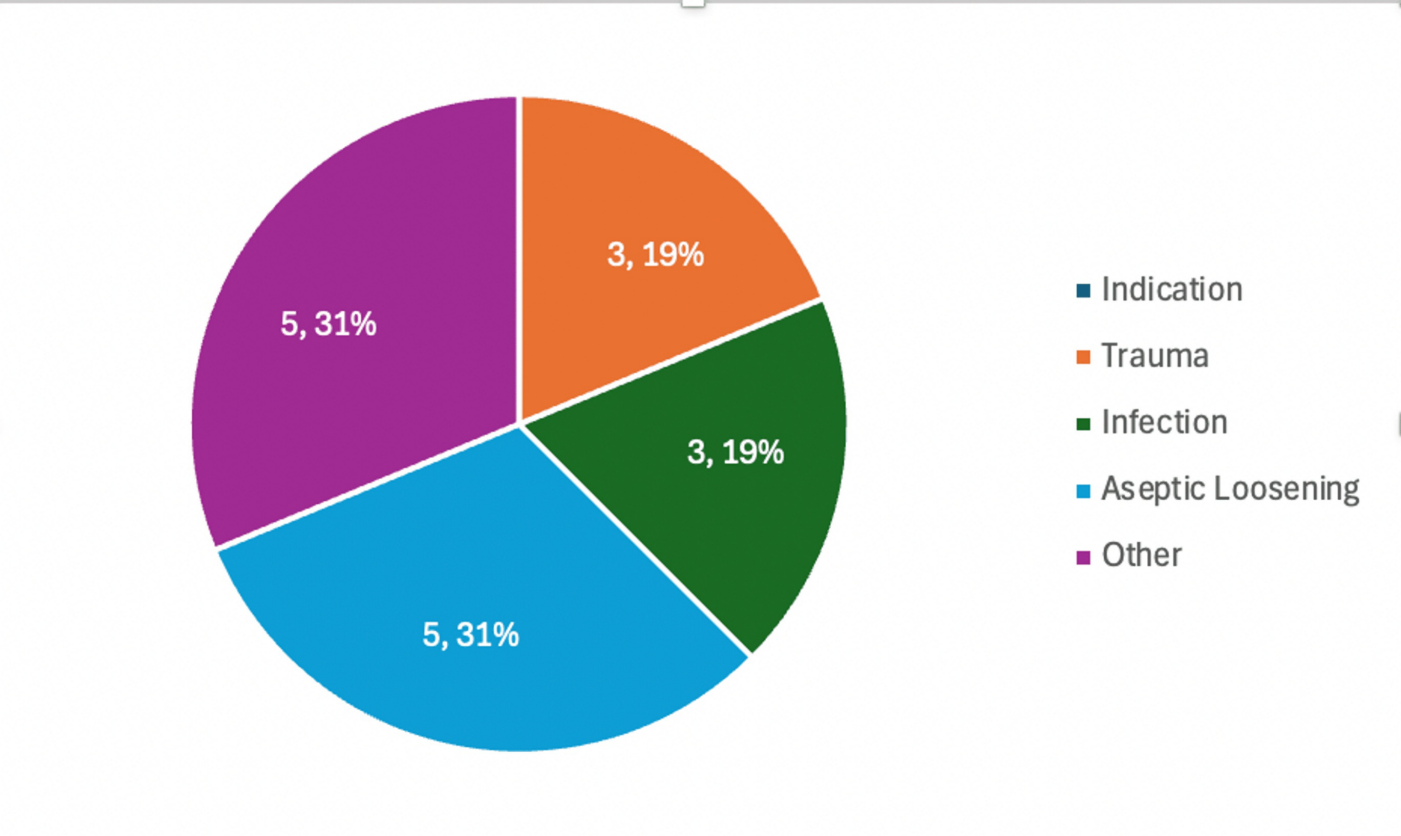
Indication For Treatment

Sixteen patients were identified from August 2018 to March 2024 with a minimum follow-up of two months (Table 2). The average age of patients was 73 years old, with a range of 49-88 years (Figure 2), with more females (62.5 %) and most operations being done on the right (75%). Most patients had a PD at the time of operation (69%). Indications for treatment included aseptic loosening (five patients), trauma (three patients) and infection (three patients) (Figure 3). These radiographic findings were, therefore, confirmed intraoperatively by a consultant within the surgical team after the initial component was removed. A minority had a hip replacement on the contralateral side (38%). According to the Charleson Comorbidity Index [8], there was a low degree of morbidity with a median CCI Score of 0.5, although the range showed some patients were more complex with the range of CCI Score being 0-8. 

**Table 2:** Cohort Characteristics CCI: Charlson Comorbidity Index

No. of patients	16	
Age (yr)		
Mean	73	
Median	76	
Range	49 -88	
Sex (no. of patients)		
Female	10	62.5%
Male	6	37.5%
Side (no. of patients)		
Right	12	75%
Left	4	25%
Pelvic discontinuity (no. of patients)	11	68.75%
Contralateral hip replacement (no. of patients)	6	37.5%
Clinical follow-up (mo)		
Mean	14	
Median	12	
Lost	1	
Died	2	
CCI Score		
Mean	1.56	
Median	0.5	
Range	0-8	
Indication		
Trauma	3	18.75%
Infection	3	18.75%
Aseptic Loosening	5	31.25%
Other	5	31.25%
Clinical Outcome		
Full Weight Bearing	10	62.5%
Mobilised With Aids	4	25%
Complication	3	18.75%
Oxford Hip Score		
Pre-Operative	15.7	
Postoperative	27.2	
Radiographic Outcome		
Integration	15	93.75%
Complication	1	6.25%

Clinical assessment 

Median clinical follow-up was 12 months, with four being actively followed up, one being lost to follow-up, and two passing away (one patient after a wound infection, the other had metastatic cancer). Sixty-three percent were full weight bearing on their last assessment, 25% were mobilising with aids, and three patients had a complication with one implant dislocating and two others suffering an infection. No fractures postoperatively were identified. Nine out of the 16 patients were contacted to assess their retrospective OHS. An average OHS of 15.7 was found preoperatively compared to 27.2 postoperatively, although this was not statistically significant (Wilcoxon Signed-Rank Test: 2.0, p: 0.07). 

Radiographic assessment 

**Figure 4:**
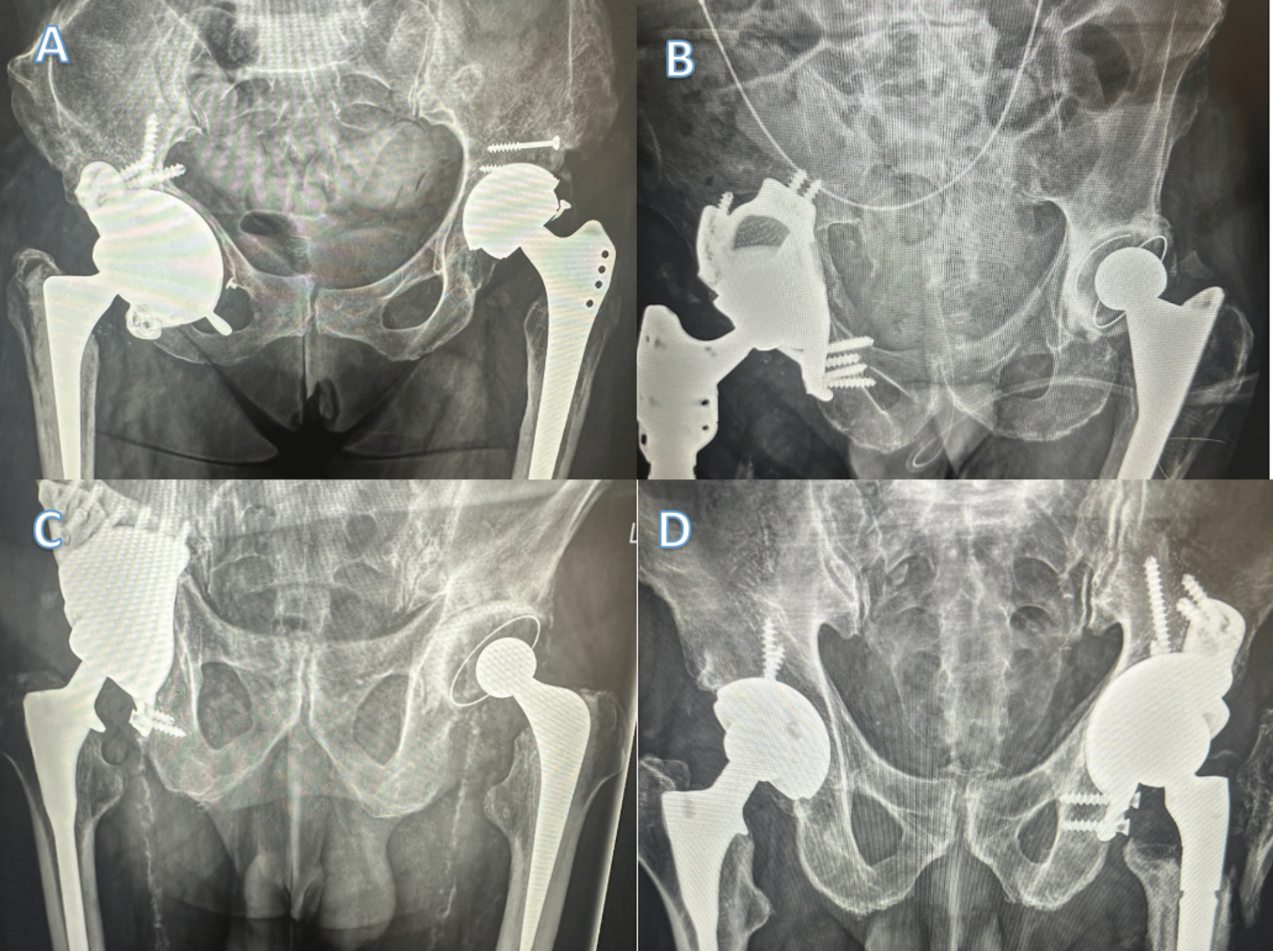
Examples of Postoperative Custom Hip Replacements A: A 56-year-old female with a background of developmental hip dysplasia had a revision of a previous right THR due to anterolateralisation of the component and acetabular bone loss. Nine-month radiographs show osteointegration. B: An 84-year-old male with a background of rheumatoid arthritis had recurrent hip infection and had a right CAI on the second-stage operation. Six-month radiographs show osteointegration. C: A 79-year-old male who had a previous road traffic accident, then had two previous right THRs due to degenerative joint disease, had a right CAI due to pain and acetabular component loosening. Six-month radiographs show osteointegration. D: An 86-year-old male had a fall onto the left THR sustaining a periprosthetic acetabular fracture with PD, thus needing a left CAI for reconstruction. Seven-month radiographs show osteointegration. THR: total hip arthroplasty; CAI: custom acetabular implant; PD: pelvic discontinuity

Postoperatively and during follow-up, radiographs were assessed for loosening, migration, and integrity alongside new bone formation at the bone-implant interface and migration (Figure 4). Satisfactory radiographs showing osteointegration were observed in 15 patients (93%), with one patient having a dislocation that couldn’t be reduced and then going to have a Girdlestone procedure. 

## Discussion

The nature of cases demonstrates the need for custom implants when in challenging cases with pelvic discontinuity or acetabular bone loss due to infection, repeat revisions and chronic instability. This conclusion is mirrored by Di Laura et al. [6], who also alluded to the poor outcomes of caces stemming from the birth of customs. Moreover, as custom implants can be performed through the original posterior-lateral approach to address pelvic discontinues, a second pelvic incision and approach is negated. Thus, reducing the risk of blood loss, neurovascular damage and infection [14].

This study demonstrates the potential for custom-made 3D implants to treat previously challenging acetabular defects, thus restoring patients' function and reducing morbidity. CT 3D reconstructions aid surgical planning by allowing the surgeon to anticipate operative challenges in positioning implants and overcome them with pre-emptive strategies [10]. These benefits have been shown to improve limb-length discrepancies and gait abnormalities even in distorted anatomy or migration of previously failed components [15].

As a Trauma Unit in the East of England, this department views these surgeries as salvage procedures where patients have limited options to improve their quality of life [16]. As demonstrated, custom implants were mainly used as the second stage procedures of an infection management pathway, aseptic loosening or trauma with PD and multiple revisions (Figure 3). In scenarios where these failed, one patient underwent a revision custom to a Lima product while the other underwent a Girdlestone, leaving limited residual mobility. Although a salvage procedure, the average age was 73, and the median CCI Score was 0.5. These figures demonstrate the need for candidates to be fit enough to survive both the anaesthetic and procedure. 

Trauma units as Colchester General Hospital's will have an Intensive Care Unit (ICU). Despite the availability of intensive care support, no patients required ICU admission postoperatively, and only one patient necessitated intraoperative vascular consultation. This practice demonstrates the ability of other trauma units to take on such cases pending the necessary surgical expertise. Moreover, managing these complex cases reduced referrals to the Major Trauma Centre and minimised their workload. 

Clinical outcomes for this study are interesting, with 63% achieving mobilisation without aids and 19% having complications. The two postoperative infections were managed with antibiotics, with one undergoing a washout, and the dislocation was treated with a Girdlestone after attempted reduction and discussion with the patient and their family. The two deaths within the cohort were in frail patients with multiple comorbidities. One patient passed away due to their metastatic transitional cell carcinoma and the other has a suppressed wound infection alongside Parkinson's disease. One patient was lost to follow-up after the six-week review but was both fully weight-bearing with satisfactory X-rays. The median follow-up was 12 months, with some patients undergoing long-term surveillance and others being discharged or having patient-initiated follow-up. Notably, the use of patient-initiated follow-up has increased recently to reduce the workload in the clinic and increase the number of new patients being seen and treated [17]. The retrospective OHS improved from 15.7 to 27.2, though this change did not reach statistical significance (Wilcoxon Signed-Rank Test: p = 0.07).

The authors conducted the assessment at least 22 months after the CAI operation. Limitations of the OHS in this context included that were that many patients had other comorbidities alongside polyarthritis, which also affected mobility. On further questioning, many noted a shortened limb postoperatively as the most debilitating factor. Notably, operative indications varied. Some patients with instability or infection were mobilising well with a high OHS, drastically different to salvage cases with PD; however, an operation was still offered using the MDT and joint patient-empowered decision-making. Therefore, combining OHS with postoperative mobility status may provide a more comprehensive assessment of functional outcomes.

Radiographic outcomes were promising, with 94% being satisfactory, demonstrating integration with no migration, loosening or fracture. Previous studies have noted small degrees of component migration when the component is press-fit until osseous ingrowth occurs [18]. However, radiographic assessment was sufficient, with no subsequent CT used unless the clinic picture suggested a complication. 

Findings on outcomes from this study match previous literature. Goriainov et al. noted good functional and 100% radiographic implant survivorship when using aMace (Materialise, Leuven, Belgium) 3D-printed tri-flange implant [19]. Within the UK colleges at Stanmore, good intermediate-term results were noted after three to six years of follow-up, with an excellent implant survival rate, good clinical outcomes, and a low complication rate [6]. However, other studies suggest custom implants can be challenging to position, leading to complications when associated with pelvic discontinuity, a finding this study doesn't support [20,21].

Limitations of this study include the retrospective nature with a limited population size in a single centre. However, considering how infrequently these operations occur, it is promising that these findings are supported by the literature. Lastly, a CT scan should have been used to assess the implants postoperatively. However, this doesn't reflect clinical practice, and such interventions would not be recommended in an economically constraining National Health Service.

## Conclusions

This study has demonstrated good functional and radiographic outcomes when using custom-made 3D-printed acetabular cups for complex acetabular defects over a six-year time horizon. These complex patients who undergo these surgeries need careful clinical and radiographical correlation with thorough surgical planning supported by the partnered company. In an NHS under pressure, follow-up should be 12 months to establish clinical and radiographical success, with subsequent follow-up being patient-initiated. Using lessons from this study, custom implants are advocated to treat complex acetabular defects and pelvic discontinuity to improve patient function and quality of life.

## Appendices

**Table 3:** Dataset of Patients Abbreviations RA – Rheumatoid Arthritis MTP & PIP – Metatarsophalangeal and Proximal Interphalangeal (joints) # – Fracture PD – Parkinson’s Disease BPH – Benign Prostatic Hyperplasia LT – Long-Term CKD – Chronic Kidney Disease T2DM – Type 2 Diabetes Mellitus HTN – Hypertension OA – Osteoarthritis DDH – Developmental Dysplasia of the Hip DJD – Degenerative Joint Disease THR – Total Hip Replacement TFR – Total Femoral Replacement DU – Duodenal Ulcer IBS – Irritable Bowel Syndrome AF – Atrial Fibrillation TCC – Transitional Cell Carcinoma FU – Follow-Up RV – Review PIFU – Patient-Initiated Follow-Up DC – Discharged OHS – Oxford Hip Score CCI – Charlson Comorbidity Index FWB – Full Weight-Bearing MTX – Methotrexate

Surgeon	Operation Date	Side	Gender	Past Medical History	Charlson Comorbidity Index (CCI)	Controlateral Hip	Indication	Indication Stratified	Pelvic Discontinuity	Outcome/Complication	Followup	F/U Mo		Radiographic Follow-Up	Notes	Age	OHS Pre	OHS Post
WARDLE	12/07/2018	L	F	RA, Schmidt's Syndrome, Addisons, MTP & PIP Repelcments, Preipos Distal Femur #	0	N	Aspectic Loosening of Acetabular Component With Limited Bone Stock	A	Y	Mobilising FWB	9 months -> RV in 6months	9		Satisfactory		77	13	40
WARDLE	06/08/2018	R	M	PD, BPH, Heaturia, LT Catheter, Pressure Sore	1	Y - cemented	Aspectic Loosening of Acetabular Component With Limited Bone Stock & Multiple Revisions	A	N	Mobilising FWB with aids, Dislocations -> Died	4/9/18 Wound Washout & Debridement -> Abx -> 6 months -> Died	N/A - Died: 02/12/21		Satisfactory		87	Na	Na
WARDLE	04/03/2019	R	F		0	N	RTC -> THR -> Revision (Jumbo Cup) & Medialisation -> Pain & Acetabular Compneent Loosening	T	Y	Mobilising FWB	57months -> DC	57		Satisfactory		69	12	41
WARDLE	15/04/2019	R	F	CKD, T2DM, HTN, Hyperlipidemia	4	N	Aspectic Loosening of Acetabular Component With Limited Bone Stock	A	Y	Mobilising FWB Stick occasionally	6 months -> 6 month RV -> Died October 2024	N/A -Lost		Satisfactory - acetabular component showing integration		88	Na	Na
SLOPER	01/03/2020	R	M	Perthes	0	N	Perthes Diseas -> OA, Poor Bone Stock & 7cm Shortenining	O	N	Mobilising FWB	6 weeks -> Lost to FU	N/A -Lost		Satisfactory (Post-Op)		57	Na	Na
WARDLE	16/03/2021	R	F	PVD, High Cholesterol, Depression, DVT	1	Y - uncemented	DDH -> DJD -> Revision as anterolateralisation of the component -> impingement of the external illiac	O	Y	Mobilising FWB	1 year -> 2 year PIFU	12		Satisfactory		49	Na	Na
WARDLE	14/04/2022	R	F	Liver Cirrhosis, Depression, R THR, Portal HTN, PCB, Hypersplenism	3	N	Dislocated R TFR, Multiple Revisions -> Oxford for TFR	O	N	Nursed in Bed -> Infection - On LT Abx	2 years -> Awaiting RV	24		Satisfactory		75	0	0
WARDLE	08/08/2022	R	F	Short Stature, Ankle Fusion, DDH Corrective Pelvic Osteotomy	0	N	DDH -> DJD	O	N	Mobilising FWB	1 year -> DC	12		Satisfactory		51	Na	Na
WARDLE	19/09/2022	L	M	Nil	0	Y -revision hip	Multiple revisions, asceptic loosening of L revision acetabular component	A	Y	Mobilising FWB	3 months -> PIFU 2 Years	3		Satisfactory		77	37	37
WARDLE	31/03/2023	R	F	RA, BL TKRs, Ankle Repalcement, DU	1	N	Infection with bone loss as part of the stage	I	Y	Mobilising FWB	1 year -> 2 year PIFU	12		Satisfactory	Done in Oxford (1st Revision - Custom) -> Revision of custom to another custom LIMA	53	20	26
LOPEZ	03/04/2023	R	F	HTN, Cataract, IBS	0	N	Acetbualr # peri-acetabular component	T	Y	Dislocated -> Underwent Girdleston 1 month later, pus found in acetabulum, therefore placed on LT IV Abx, mobilising via Hoist on ward.	On Ward	14		Dislocated -> Girdlestone		85	0	0
LOPEZ	12/05/2023	L	M	HTN, AF, CKD, TCC- Metastatic	8	N	Acetabular bone loss & dislocation	O	Y	Mobilising with Frame -> Passed Away	6 weeks -> Passed Away	N/A - Died 01/02/24		Satisfactory - acetabular component showing intergration		88	Na	Na
WARDLE	05/06/2023	R	F	Hypothyroidism, Bowel Ca, Cataracts	6	Y - cemented	Aspectic Loosening of Acetabular Component & Pelvic Discontinuity	A	Y	Mobilising FWB	7 weeks -> Lost to FU?	N/A -Lost		Satisfactory - acetabular component showing intergration		85	Na	Na
LOPEZ	04/08/2023	L	M	Seroneg Arthropathy	0	Y - cemented	Infected Loose THR Stage used custom	I	Y	Mobilising FWB, needs physio input, Admitted for hand swelling (On Mtx)	5 months -> TBC 3 months	5		Satisfactory - acetabular component showing iintergration		76	12	37
SLOPER	05/10/2023	R	M	HTN, High chol, TIA	1	Y - uncemented	Infected Loose THR (Dsilocated) Stage: Stage used custom	I	N	Mobilising FWB Stick occasionally	6 months -> TBC 6mnths	6		Satisfactory - acetabular component showing intergration		80	47	40
SLOPER	21/03/2024	R	F	HTN, Sleep Apnoea, Anxiety, Depression, Hypothyroidism	0	N	Periprosthetic Acetabular # -> Displaced	T	Y	Mobilising FWB with aids	2 months -> TBC 2 mnths	2		Satisfactory - acetabular # healing		75	0	24
